# Complete genome sequence of *Brucella abortus* isolated from a human blood culture sample in Tanzania

**DOI:** 10.1128/mra.00930-23

**Published:** 2024-01-30

**Authors:** Gershom A. Mbwambo, Marco van Zwetselaar, Tolbert Sonda, AbdulHamid S. Lukambagire, Judith S. Njau, Boaz Wadugu, Ignass P. Ignass, Nelson B. Amani, Ephrasia A. Hugho, Matthew P. Rubach, Philoteus Sakasaka, Rose S. Oisso, Nestory Mkenda, Gabriel Shirima, Roland T. Ashford, Daniel T. Haydon, Venance P. Maro, Rudovick R. Kazwala, Happiness H. Kumburu, Blandina T. Mmbaga, Jo E. B. Halliday

**Affiliations:** 1 Kilimanjaro Clinical Research Institute, Moshi, Tanzania; 2 EcoHealth Alliance, New York, New York, USA; 3 Institute of Public Health, Kilimanjaro Christian Medical University College, Moshi, Tanzania; 4 Department of Medicine, Division of Infectious Disease and International Health, Duke Global Health Institute, Duke University School of Medicine, Durham, North Carolina, USA; 5 Endulen Hospital, Arusha, Tanzania; 6 Nelson Mandela Africa Institute of Science and Technology, Arusha, Tanzania; 7 Department of Bacteriology, Animal and Plant Health Agency, Weybridge, United Kingdom; 8 School of Biodiversity, One Health & Veterinary Medicine, College of Medical Veterinary and Life Sciences, University of Glasgow, Glasgow, United Kingdom; 9 Department of Internal Medicine, Kilimanjaro Christian Medical University College, Moshi, Tanzania; 10 Department of Veterinary Medicine and Public Health, College of Veterinary Medicine and Biomedical Sciences, Sokoine University of Agriculture, Morogoro, Tanzania; 11 Kilimanjaro Christian Medical University College, Moshi, Tanzania; 12 Kilimanjaro Christian Medical Centre, Moshi, Tanzania; University of Maryland School of Medicine, USA

**Keywords:** *Brucella*, molecular epidemiology, zoonoses

## Abstract

*Brucella abortus* causes infections in humans and livestock. Bacterial isolates are challenging to obtain, and very little is known about the genomic epidemiology of this species in Africa. Here, we report the complete genome sequence of a *Brucella abortus* isolate cultured from a febrile human in northern Tanzania.

## ANNOUNCEMENT

Brucellosis is a zoonosis caused by bacteria of the genus *Brucella. Brucella abortus* causes human illness and production impacts in livestock. *Brucella* species are challenging to isolate. Culture involves considerable infection risk for laboratory personnel, and high containment laboratory facilities are required for safe manipulation of isolates (1). *Brucella abortus* is endemic in Tanzania and has been isolated previously (2). Despite widespread distribution across Africa, there are very few published sequences of *Brucella abortus* from Africa and none from Tanzania specifically. We report a complete genome sequence of *Brucella abortus* isolated from a febrile human who presented for care in northern Tanzania in 2017 (3). A blood sample was collected and inoculated into Castañeda media (prepared at the Animal and Plant Health Agency Weybridge, UK). Culture bottles were incubated in a CO_2_ incubator at 5%–10% CO_2_ and 37°C. Bottles were examined for growth every 72 hours for up to 35 days. Isolates of Gram-negative *coccobacilli* with positive reactions for urease, catalase, and oxidase were classified as presumptive *Brucella* spp. and stored on Microbank beads (Pro-Lab Diagnostics, Bromborough, UK) at −70°C (3). Ethical approval for the study was granted by the Kilimanjaro Christian Medical Centre Ethics Committee (698), National Institute of Medical Research (NIMR), Tanzania (NIMR/HQ/R.8c/Vol. I/1140), and University of Glasgow College of Medical, Veterinary and Life Sciences Ethics Committee (200140149).

Isolate manipulation (culture and extraction) was done at the Kilimanjaro Clinical Research Institute-Biotechnology Laboratory. The thawed isolate was plated on sheep blood agar, incubated at 37°C and 5% CO_2_ and observed for 2 days before selection of pure colonies morphologically consistent with *Brucella*. Extraction of genomic DNA was performed using the Quick-DNA HMW MagBead Kit (D6060, Zymo) and the QIAamp DNA Mini Kit (51306, Qiagen). Genomic DNA from both kits was assessed for quantity using a Qubit 4.0 fluorometer and for quality using a Nanodrop spectrophotometer. The input concentration was adjusted to approximately 53 ng/µL for MinION and 2.0 ng/µL for Illumina sequencing, respectively. Long reads were generated using a MinION Mk1B sequencer and R9.4.1 flow cell (Oxford Nanopore Technologies) after library preparation using the Rapid Barcoding Sequencing kit (SQK-RBK004, Oxford Nanopore Technologies). Short reads were generated using the Illumina Miseq sequencer and 2 × 250 bp paired-end protocol after library preparation using the Illumina Nextera XT protocol (Illumina, Inc.). Long reads were basecalled and demultiplexed with Guppy (v6.4.2) using the “sup” model. Long-read assembly was done with Flye (v2.9.2-16) in “nano-hq” configuration using five polishing iterations (4). The resulting draft assembly consisted of two complete circular contigs and was polished with Medaka (v1.9.1) using the “r941_min_sup_g507” model (5). Short reads were then used to polish the long-read assembly using Polypolish (v0.5.0) with default settings. The resulting complete genome assembly was annotated with Prokka (v1.14.6) and EMBLmyGFF3 (v2.3)(6). Table 1 gives details of the sequence obtained.

**TABLE 1:** Characteristics of the *B. abortus* sequence

Characteristic	Isolate data
Study ID	PRJEB66434
Year of isolation	2017
Country of origin	Tanzania
Source	Human blood culture
Number of ONT reads	66,790
ONT average read length (bp)	7,849
Number of sequenced bp, ONT	524,253,445
Genome coverage, ONT	160×
Number of Illumina reads (pairs)	251,458
Number of sequenced bp, Illumina	107,559,286
Genome coverage, Illumina	33×
Genome size after hybrid assembly (bp)	3,280,926 (2,118,235 + 1,162,691)
GC content after hybrid assembly (%)	57.2%
Number of genes	3,238
Number of CDS	3,115
Number of rRNA/tRNA/tmRNA/ncRNA	9/54/1/59
MLST	ST32
Genome accession number	GCA_963555505

## Data Availability

This project has been deposited in the European Nucleotide Archive under accession number PRJEB66434. Raw reads are under accessions ERR12080966 (ONT) and ERR12080967 (MiSeq). The genome assembly has GenBank accession GCA_963555505 and chromosome accessions OY741352 and OY741353.
